# Comparison of choroidal biomarkers and scleral thickness in keratoconus in relation to corneal cross-linking

**DOI:** 10.1007/s00417-026-07243-y

**Published:** 2026-04-24

**Authors:** Nicola Valsecchi, Giulia Folgaria, Simone Febbraro, Andrea Lorenzi, Samir Shhada, Mohammed Abdul Rasheed, Natalie di Geronimo, Antonio Moramarco, Jay Chhablani, Luigi Fontana, Maurizio Mete

**Affiliations:** 1 Ophthalmology Unit, Dipartimento di Scienze Mediche e Chirurgiche, Alma Mater Studiorum University of Bologna, Bologna, Italy; 2 IRCCS Azienda Ospedaliero-Universitaria di Bologna, Bologna, Italy; 3 Eye Clinic, Santa Maria delle Croci Hospital, Ravenna, Italy; 4 Department of Ophthalmology, School of Medicine, University of Pittsburgh, Pittsburgh, PA USA

**Keywords:** Keratoconus, Cross-linking, Pachychoroid, Multimodal imaging, Choroidal vascularity index, Artificial intelligence

## Abstract

**Purpose:**

To investigate whether corneal collagen cross-linking (CXL) is associated with measurable remodeling of extra-corneal tissues in KC, by comparing choroidal and scleral structural biomarkers between treatment-naïve and CXL-treated eyes.

**Methods:**

This is a single center, cross-sectional, observational study. Anterior scleral thickness (ST) was quantified using swept-source anterior segment optical coherence tomography (AS-OCT; CASIA2, Tomey, Japan). Ocular biomechanical parameters were measured with the Ocular Response Analyzer (ORA; Reichert, Germany). Subfoveal choroidal thickness (SFCT), peripapillary choroidal thickness (PPCT) were assessed with spectral-domain OCT (SPECTRALIS HRA-OCT, Heidelberg Engineering, Germany). Choroidal vascularity index (CVI) and total choroidal area (TCA) were computed using a validated semi-automated algorithm. A subgroup analysis compared treated and untreated fellow eyes in unilateral CXL cases.

**Results:**

65 keratoconic eyes of 65 patients were recruited – 35 treatment-naïve *versus* 30 post–epithelium-off cross-linking (≥ 6 months post-treatment). CXL-treated eyes exhibited significantly greater Sub-Foveal Choroidal Thickness (SFCT) (421.1 ± 81.9 μm vs. 352.9 ± 81.9 μm; *p* = 0.016), mean Peripapillary Choroidal Thickness (PPCT) (238.0 ± 72.9 μm vs. 199.5 ± 65.3 μm; *p* = 0.038), and mean Total Choroidal Area (TCA) (1.45 ± 0.32 mm² vs. 1.29 ± 0.27 mm²; *p* = 0.046) compared with treatment-naïve eyes, whereas Choroidal Vascularity Index (CVI) and Scleral Thickness (ST) showed no significant differences (*p* > 0.05). In unilateral cases, treated eyes demonstrated consistent increases in SFCT, TCA, and PPCT relative to fellow untreated eyes (*p* < 0.05). PPE alterations occurred exclusively in the CXL group (16.7%). Multivariate regression analysis identified previous CXL as the only independent factor associated with increased SFCT (β = 0.340, *p* = 0.040).

**Conclusions:**

CXL appears to be associated with thicker choroids in keratoconic eyes, independent of biomechanical or scleral thickness variations. These findings support the hypothesis that CXL might be linked to subclinical remodeling of deeper ocular layers, potentially affecting choroidal-scleral fluid dynamics. Further prospective longitudinal studies with larger cohorts and multimodal assessment are warranted to clarify the mechanisms, time course, and long-term implications of choroidal changes following CXL.

**Supplementary Information:**

The online version contains supplementary material available at https://doi.org/10.1007/s00417-026-07243-y.

## Key Messages

*****What is known***:**

- Recent studies have demonstrated a significant increase in choroidal thickness in eyes with Keratoconus (KC);
- The potential pathophysiological relationship between KC and pachychoroid spectrum is a topic of ongoing discussion;
- Increased scleral thickness, a characteristic feature of Pachychoroid Disease Spectrum (PDS) may serve as a predictive biomarker for Pachychoroid Pigment Epitheliopathy (PPE) in patients with KC;
- Previous studies have shown that cross-linking (CXL) can induce biomechanical and ultrastructural changes in the sclera, notably increasing scleral stiffness and reducing permeability.

*****What is new***:**

- Measurable and significant differences were found between untreated and CXL-treated keratoconic eyes, with the latter showing increased choroidal thickness;
- PPE-like changes were observed exclusively in CXL-treated eyes, and prior-CXL was found to be the only significant predictor of choroidal thickness in this model;
- CXL may be associated with pachychoroid alterations in keratoconic eyes.

**Supplementary Information:**

The online version contains supplementary material available at https://doi.org/10.1007/s00417-026-07243-y.

## Introduction

Keratoconus (KC) is a bilateral, progressive ectatic disorder of the cornea, characterized by stromal thinning, conical protrusion, and irregular astigmatism [1]. Although its precise pathogenesis remains unclear, KC is widely regarded as a multifactorial disease, characterized by structural alterations in collagen organization and disruptions to the extracellular matrix (ECM). The ECM is critical not only for maintaining the biomechanical stability of the corneoscleral envelope but also constitutes a fundamental component of the walls of retinal and choroidal vasculature [2].

Recent studies have demonstrated a significant increase in choroidal thickness in eyes with KC, prompting investigation into a potential pathophysiological relationship between KC and the pachychoroid spectrum disorders [3–8]. Eandi et al. originally hypothesized that KC may confer increased susceptibility to central serous chorioretinopathy (CSC) [9]. In 2024, Feo et al. identified pachychoroid pigment epitheliopathy (PPE), an early manifestation of CSC, in almost 20% of KC patients [10]. Furthermore, Forte et al. recently reported that increased scleral thickness, a characteristic feature of PDS, may serve as a predictive biomarker for PPE in patients with keratoconus [11].

Corneal collagen cross-linking (CXL) represents the gold standard treatment for halting KC progression by strengthening and stabilizing stromal collagen lamellae [12]. Beyond its corneal effects, both in vivo human studies and animal models have demonstrated that CXL may induce biomechanical and ultrastructural changes in the sclera, notably increasing scleral stiffness and reducing permeability, particularly in the equatorial regions compared to the posterior sclera [13, 14].

To date, data regarding choroidal and scleral remodeling induced by CXL are limited. Elucidating these changes may provide critical insights into the extra-corneal effects of CXL. Accordingly, the present study aimed to evaluate anterior scleral thickness, choroidal thickness, and ocular biomechanical properties in patients with KC by comparing eyes with and without prior CXL treatment.

## Materials and methods

### Study design and population

This retrospective study was conducted at Unit of Ophthalmology of University of Bologna, where consecutive patients with KC were recruited between January 2025 and June 2025. All procedures adhered to the principles of the Declaration of Helsinki, and all participants provided written informed consent. This study was approved by the Ethics Committee of Bologna, Italy (Cod CE: 53/2025/Oss/AOUBo).

Patients over 16 years of age with a confirmed diagnosis of KC were included in the study. Eyes were classified as treatment-naïve if there was no prior history of ocular surgery. Conversely, eyes designated as previously treated with CXL had undergone only accelerated, epithelium-off CXL at least six months before imaging acquisition [15]. To avoid inter-eye correlation bias, only one eye per patient was included in the analysis; when both eyes met the inclusion criteria, the right eye was systematically selected for statistical evaluation to ensure a standardized selection process and to avoid investigator-dependent eye selection. Furthermore, comparisons were made between the treated and untreated eyes of patients who underwent unilateral CXL to evaluate differences in choroidal thickness, scleral thickness, and biomechanical properties.

We excluded eyes with the following characteristics: axial length < 21 or > 27 mm, history of other chorioretinal or optic nerve disorders, any history or active signs of scleritis or episcleritis, relevant optical media opacities or insufficient fixation not allowing high quality imaging, eyes which underwent previous corneal transplantation or other ocular surgical procedures, current steroid treatment, systemic diseases who could exert a possible impact on choroidal or scleral thickness such as inflammatory conditions, and current pregnancy or history of pregnancy in the previous 12 months.

### Ophthalmological assessment

Past general medical history along with past ocular history was collected. Each patient underwent an ophthalmological assessment which included best corrected visual acuity (BCVA) measurement, slit lamp biomicroscopy examination, intraocular pression (IOP) measurement using the Ocular Response Analyzer (ORA) (Reichert Ophthalmic Instruments, Depew, NY), axial length measurement (IOLMaster 700; Carl Zeiss Meditec, Jena, Germany), corneal tomography (Pentacam HR; Oculus, Wetzlar, Germany), spectral domain optical coherence tomography (SD-OCT) imaging (SPECTRALIS HRA-OCT; Heidelberg Engineering, Heidelberg, Germany) and anterior segment OCT (AS-OCT) (CASIA2, TOMEY) for anterior scleral measurements. Staging for KC was assessed using the Amsler-Krumeich classification [16].

### Ocular response analyzer measurements

Corneal hysteresis (CH), corneal resistance factor (CRF), Goldmann-related IOP (IOPg), and corneal-compensated IOP (IOPcc) were assessed using the ORA device, which allows for their calculation by measuring the difference between two applanation pressure events induced by a metered air puls [17]. The ORA examinations were performed by two operators (GF, AL) unaware of the patient’s characteristics at the time of presentation; each exam was conducted at the same day time (*10 am – 12 pm*) to avoid potential interfering effects of diurnal IOP fluctuations. To obtain central corneal thickness (CCT)- adjusted readings, the CCT values were calculated using the Pentacam HR (Oculus, Wetzlar, Germany). The best measurement from 4 recordings with a waveform score > 7 was used for analysis.

### Anterior scleral measurements

**Fig. 1:**
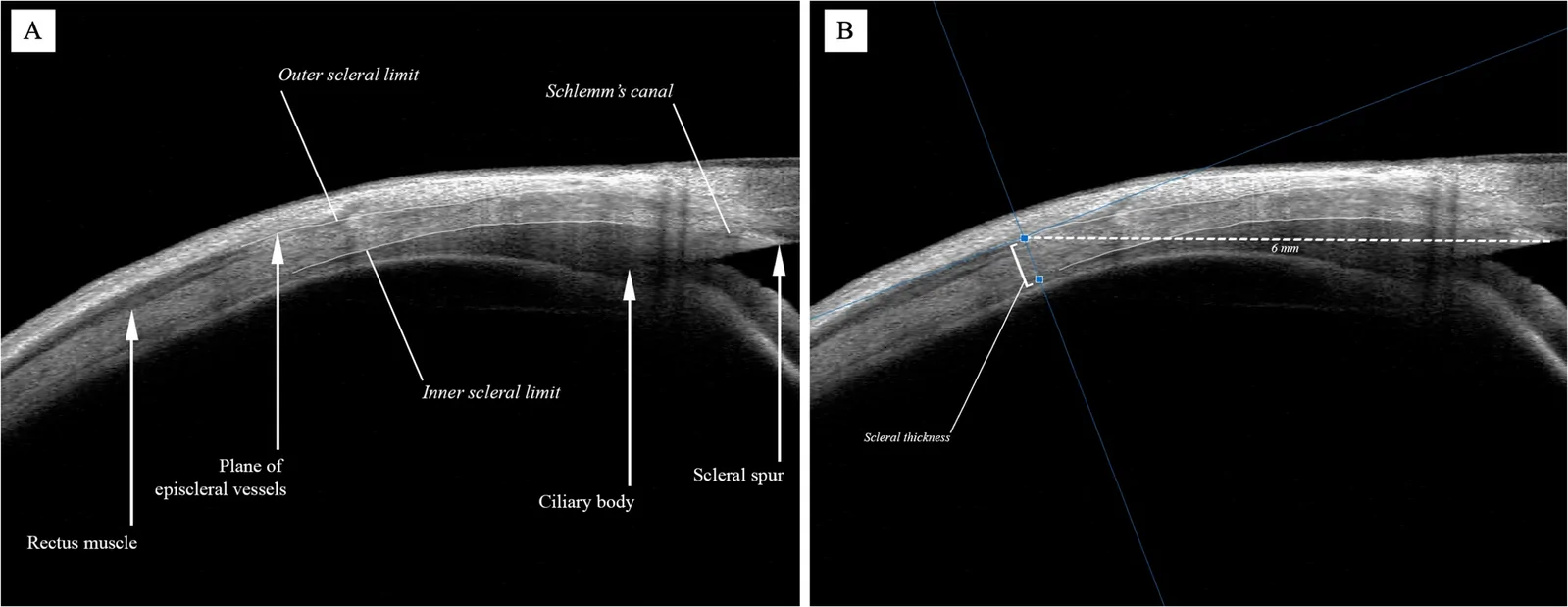
Anterior segment OCT showing measurement of scleral thickness. (**A**) Anatomical landmarks: scleral spur, ciliary body, Schlemm’s canal, rectus muscle, episcleral plane, and scleral boundaries. (**B**) Scleral thickness was measured 6 mm posterior to the scleral spur

Anterior segment cross-sectional images were acquired with swept source AS-OCT (CASIA2; TOMEY) using the same method described in previous study [18]. The examinations were conducted by two operators, both blind to subject’s characteristics (GF, AL). Before each acquisition, by gazing in the opposite direction of the muscle targeted, the 4 recti muscles were firstly detected as low reflective bands adjacent to the anterior sclera. The AS-OCT scans were acquired and collected parallel with each muscle with raster scan mode (16B scans, 4 mm width). After each acquisition, the following anatomical landmarks were identified: scleral spur (1), outer scleral limit (2), inner scleral limit (3). Scleral thickness was manually measured on the same device with the caliper function 6 mm posterior to the scleral spur perpendicularly to a line parallel to the inner scleral wall at the 4 locations to acquire *superior*, *inferior*, *nasal*, and *temporal* values in millimeters. Measurements were performed by two independent observers (G.F, A.L), with discrepancies resolved by a third observer (MM). See Fig. 1.

### Subfoveal and peripapillary choroidal thickness measurements

All subjects underwent the same SD-OCT protocol with the Heidelberg Spectralis device (Heidelberg Engineering, Germany). Scans were acquired between 10:00 AM and 12:00 PM to minimize diurnal variation. For macular evaluation, a high-resolution 20° × 15° raster scan consisting of 145 B-scans spaced 30 μm apart was obtained, along with single vertical and horizontal scans centered on the fovea. Each scan had an automatic real-time (ART) value of 14 or higher. Subfoveal choroidal thickness (SFCT) was manually measured as the vertical distance from the hyperreflective Bruch’s membrane to the inner scleral surface using the built-in caliper tool. Peripapillary choroidal thickness (PPCT) was assessed using a standard retinal nerve fiber layer (RNFL) protocol, which involved a 360-degree, 3.4 mm diameter peripapillary circle scan, as previously described [19]. ^,^ [20] The PPCT was measured manually from the hyperreflective line corresponding to the retinal pigment epithelium (RPE) to the inner surface of the sclera, at the temporal, superior, nasal, and inferior segments. Measurements were performed by two independent observers (G.F, A.L), with discrepancies resolved by a third observer (N.V). The presence of PPE was defined in binary terms (present or absent) determined by the detection of RPE abnormalities and enlarged choroidal vessels on OCT imaging, supported by characteristic features observed on fundus autofluorescence. The assessment was conducted by two observers (NV, MM), with discrepancies resolved by a third observer (JC).

### Choroidal vascularity index analysis

**Fig. 2:**
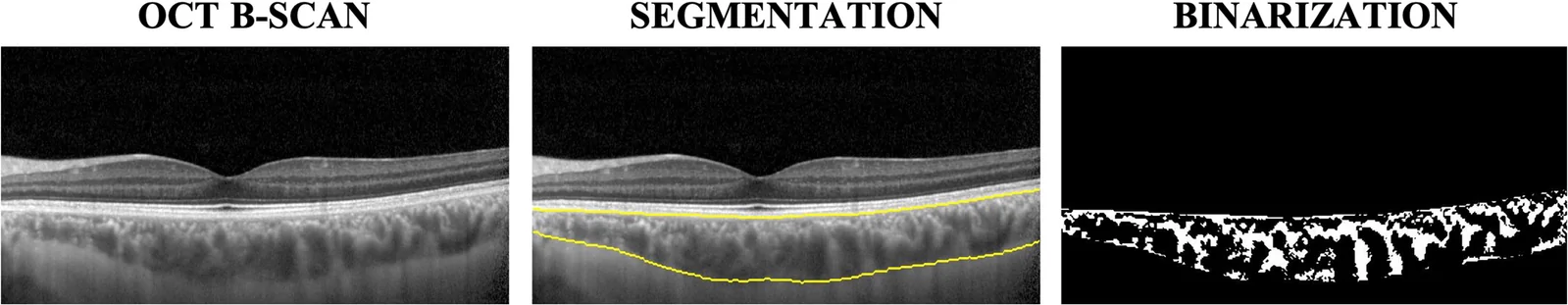
Artificial intelligence-based automated algorithm for choroidal vascularity index (CVI) calculation. Bright areas represent stromal choroid, dark areas luminal choroid; CVI is the ratio of luminal area to total choroidal area

To assess the choroidal vascularity index (CVI), SD-OCT (Heidelberg Engineering, Heidelberg, Germany) images were used. CVI was measured using an artificial intelligence-based automated algorithm that included shadow compensation, denoising, localization of the inner and outer choroidal boundaries, and segmentation of the choroidal region from the rest of the scan, as previously described [21]. Binarization of the choroid generated bright regions labeled as stromal choroidal areas (SCA) and dark regions labeled as luminal choroidal areas (LCA). See Fig. 2. CVI was calculated as the ratio of the luminal choroidal area to the total choroidal area (TCA).

### Statistical analysis

Demographic and clinical data were summarized using descriptive statistics, including measures of precision and variability, based on the type of variable (categorical or continuous). Normality in the distribution of continuous data was assessed using the Shapiro–Wilk test, and parametric tests were applied for the analysis. The Fisher’s exact test was used to examine associations between categorical variables. A T-test was employed to compare demographic data, biomechanical properties, scleral thickness, and choroidal biomarkers between eyes that had undergone CXL and those that had not. Inter-rater reliability for SFCT and PPCT measurements was assessed using the absolute agreement model of the intraclass correlation coefficient (ICC). A multivariate regression analysis was performed to investigate the association between SFCT, PPCT and TCA and the following set of predictor variables: age, previous CXL, AL, KC stage, CH, and ST, selected based on their potential influence on choroidal biomarkers. Multicollinearity between predictors was assessed prior to their inclusion in our regression model. Also, linear mixed models were used for comparisons between the treated and untreated eyes of patients who underwent unilateral CXL. P-values < 0.05 were considered statistically significant. All statistical analyses were conducted using IBM SPSS Statistics version 26 (IBM, Chicago, IL, USA).

## Results

### Demographic data

A total of 65 eyes of 65 patients were included in the study. Among them, 35 eyes were treatment naïve, and 30 eyes received previous epi-off CXL 20.12 ± 8.16 months prior to ophthalmological assessment. The two groups were comparable in terms of age and sex distribution. Other baseline characteristics at presentation including BCVA, K(mean), AL, IOPg, IOPcc, CH and CRF did not significantly differ between the two groups, except for pachymetry values. No differences in terms of ocular biomechanics were observed. See Table 1.

**Table 1:** Demographic data are reported

	Treatment-naive(n=35)	Previous CXL(n=30)	P value
Age, mean ± SD	22.4 ± 5.77	23.72 ± 5.26	0.467
Sex female, number (%)	8 (22.8%)	7 (23.3%)	0.674
Staging, number (%)			
- 1	22 (62.8%)	18 (60%)	
- 2	11 (31.4%)	7 (23.3%)	
- 3	2 (5.7%)	5 (16.6%)	
BCVA, mean ± SD (LogMar)	0.62 ± 0.31	0.53 ± 0.31	0.369
IOPg, mean ± SD (mmHg)	10.26 ± 2.86	10.33 ± 2.99	0.939
IOPcc, mean ± SD (mmHg)	13.45 ± 3.47	13.99 ± 2.39	0.394
CH, mean ± SD	8.86 ± 2.01	8.13 ± 1.57	0.230
CRF, mean ± SD	7.54 ± 1.81	6.93 ± 1.91	0.318
Axial length, mean ± SD (mm)	24.15 ± 1.47	23.86 ± 0.67	0.451
Pachymetry, mean ± SD (µm)	499.05 ± 31.34	460.67 ± 36.72	0.001
K mean, mean ± SD (D)	46.74 ± 3.47	47.42 ± 4.17	0.536

*CXL *cross-linking, *SD* standard deviation, *BCVA *best corrected visual acuity, *IOPg *Goldmann intraocular pressure, *IOPcc *corneal compensated intraocular pressure, *CH *corneal hysteresis, *CRF* corneal resistance factor *K mean* mean keratometry

### Choroidal biomarkers and scleral thickness among the two groups

**Fig. 3:**
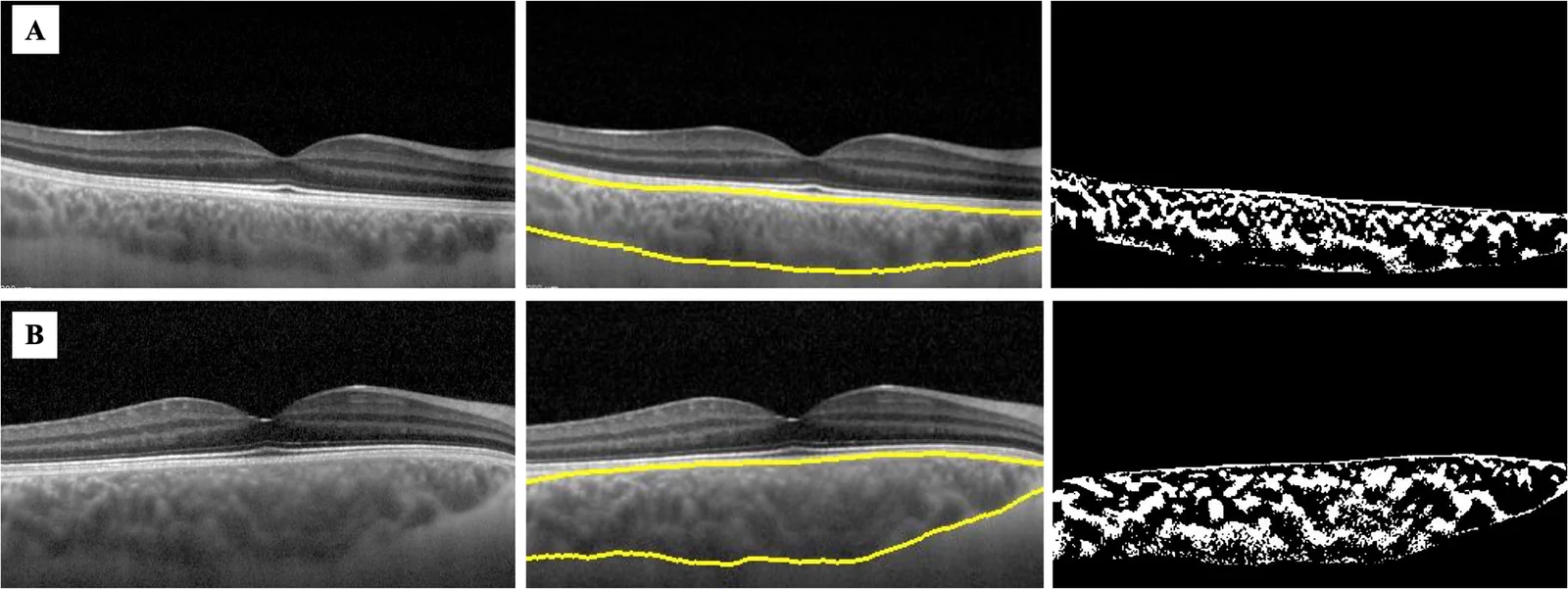
Optical coherence tomography scans with choroidal binarization in (**A**) treatment-naïve and (**B**) cross-linking (CXL)-treated keratoconic eye. Note the increased total choroidal area in the eye with previous CXL

Compared to treatment-naïve eyes, eyes that had received previous epi-off CXL presented significantly increased SFCT (421.11 ± 81.93 vs. 352.89 ± 81.9 μm; *p* = 0.016), mean PPCT (237.99 ± 72.92 *versus* 199.51 ± 65.26 μm; *p* = 0.038), inferior PPCT (220.67 ± 100.97 *versus* 167.6 ± 58.41 μm; *p* = 0.032), and nasal PPCT (249.61 ± 79.28 *versus* 194.5 ± 66.73 μm; *p* = 0.016). Additionally, we found statistically significant differences in TCA and LCA in eyes that had received previous epi-off CXL compared to those that had not (*p* = 0.046, *p* = 0.036, respectively). See Fig. 3. On the other hand, no significant differences were observed in terms of ST and CVI. See Table 2. Overall, both graders showed a good level of agreement in measuring SFCT and PPCT values (ICC = 0.907, CI 0.875–0.953).

**Table 2:** Choroidal thickness, scleral thickness and choroidal biomarkers among the two groups examined

	Treatment-naïve(n=35)	Previous CXL(n=30)	P value
Macular choroidal parameters			
SFCT, mean ± SD (µm)	352.89 ± 81.9	421.11 ± 81.93	0.016
TCA, mean ± SD (mm^2^)	1.29 ± 0.27	1.45 ± 0.32	0.046
SCA, mean ± SD (mm^2^)	0.49 ± 0.15	0.53 ± 0.13	0.396
LCA, mean ± SD (mm^2^)	0.81 ± 0.14	0.93 ± 0.20	0.036
CVI, mean ± SD (%)	0.63 ± 0.05	0.64 ±0.03	0.522
Peripapillary choroidal thickness			
Mean PPCT, mean ± SD (µm)	199.51 ± 65.26	237.99 ± 72.92	0.038
Inferior PPCT, mean ± SD (µm)	167.6 ± 58.41	220.67 ± 100.97	0.032
Superior PPCT, mean ± SD (µm)	207.3 ± 82.01	251.72 ± 93.58	0.128
Nasal PPCT, mean ± SD (µm)	194.5 ± 66.73	249.61 ± 79.28	0.016
Temporal PPCT, mean ± SD (µm)	215.3 ± 85.9	246.78 ± 69.18	0.225
Scleral thickness			
Mean, mean ± SD (mm)	0.47 ± 0.03	0.48 ± 0.04	0.344
Inferior, mean ± SD (mm)	0.48 ± 0.06	0.5 ± 0.04	0.329
Superior, mean ± SD (mm)	0.45 ± 0.05	0.47 ± 0.05	0.164
Nasal, mean ± SD (mm)	0.47 ± 0.03	0.47 ± 0.05	0.495
Temporal, mean ± SD (mm)	0.47 ± 0.04	0.47 ± 0.05	0.953

*CXL *cross-linking, *SD *standard deviation, *SFCT *subfoveal choroidal thickness, *TCA *total choroidal area, *SCA *stromalchoroidal area, *LCA *luminal choroidal area, *CVI *choroidal vascularity index, *PPCT *peripapillary choroidal thickness

**Fig. 4:**
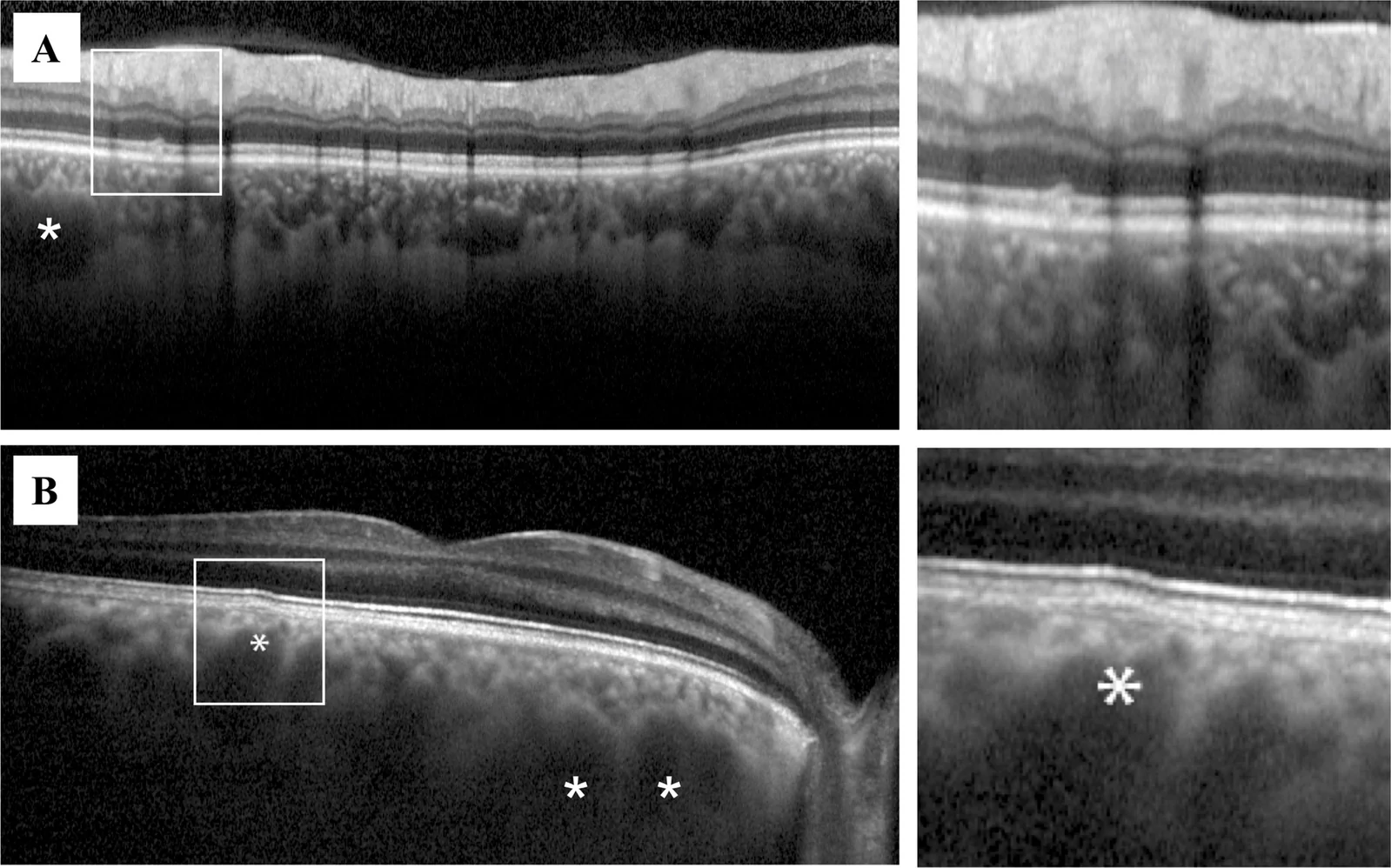
Optical coherence tomography (OCT) scans of two keratoconic eyes that showed features compatible with pachychoroid pigment epiteliopathy. (**a**) Peripapillary retinal pigment epithelium (RPE) hyperplasia with pachyvessels (white asterisk) in a 32-year-old. (**b**) Macular RPE thickening above enlarged Haller layer vessels (white asterisks) in a 26-year-old male

Additionally, 5 eyes (7.69%) showed changes consistent with PPE, and these cases were observed exclusively within the cross-linked keratoconus group (5/30, 16.66%). In 3 cases, alterations were observed in the peripapillary region, whereas in 2 cases alterations were observed in the macula. All PPE cases were asymptomatic and identified incidentally, with no signs of advanced pachychoroid spectrum disorders. Representative examples are shown in Fig. 4. To evaluate whether the selection of the right eye influenced the results, we performed a sensitivity check by including the left eye in patients for whom both eyes met the inclusion criteria (12 eyes in the treatment-naïve group, 10 in the CXL group), observing similar results. See Supplementary Table 1.

*Choroidal biomarkers and scleral thickness among treated and untreated eyes in patients who underwent unilateral corneal cross-linking*.

**Fig. 5:**
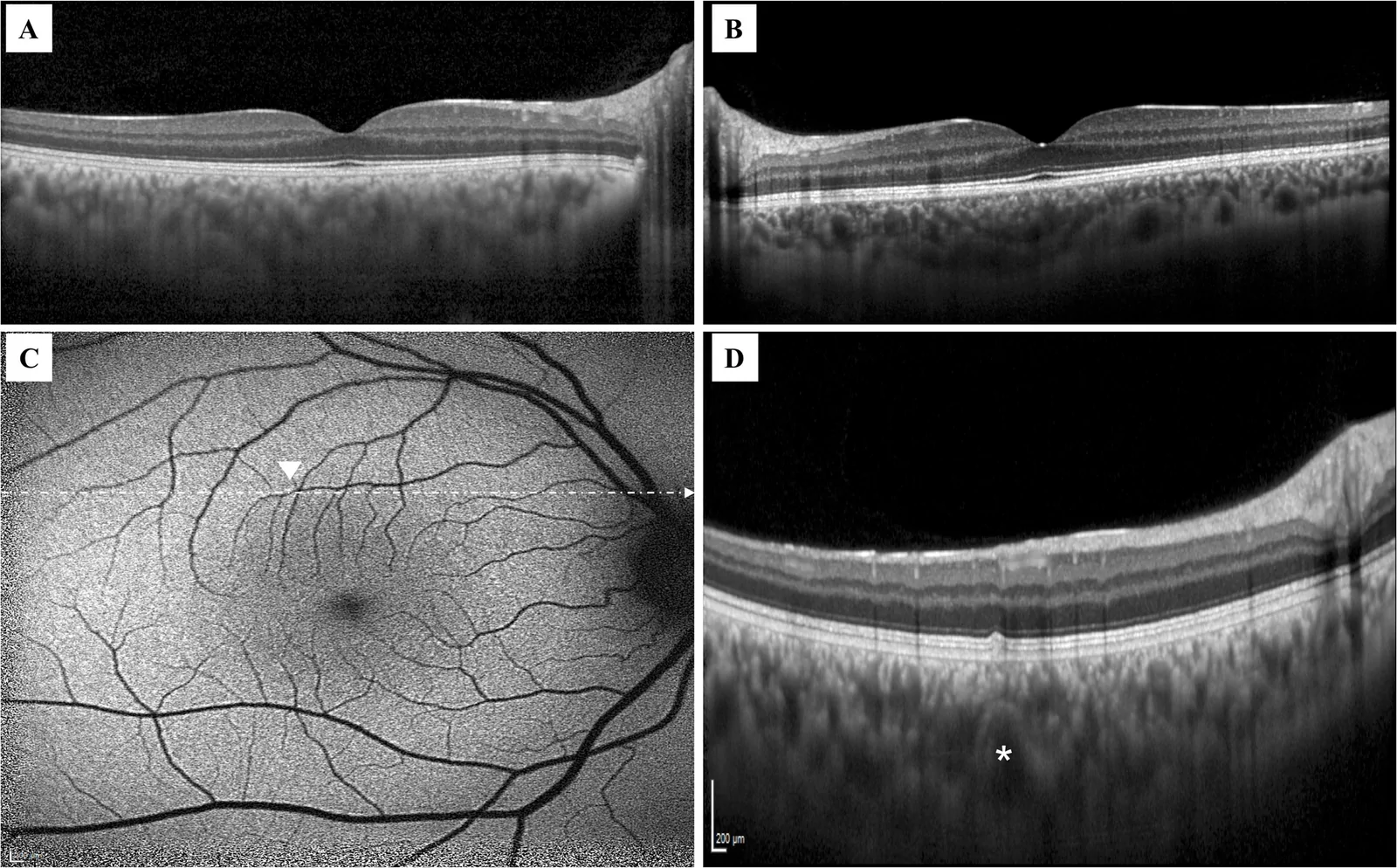
Optical coherence tomography (OCT) scans of a patient with keratoconus who underwent unilateral corneal cross-linking (CXL). (**a**) Increased choroidal thickness in the CXL-treated right eye (7 months post-treatment). (**b**) Fellow untreated left eye shows reduced choroidal thickness. (**c**) Fundus autofluorescence of the right eye reveals hyperautofluorescence (arrowhead). (**d**) Corresponding retinal pigment epithelium hyperplasia over a pachyvessel in Haller’s layer (white asterisks)

Among the 30 patients in the CXL group, 20 received unilateral treatment. Mean age of this cohort was 23.71 ± 5.08 years, and 25% were females. BCVA was 0.65 ± 0.30 and 0.54 ± 0.33 in untreated and treated eyes, respectively (*p* = 0.191). Also, no differences in KC stage and K mean were observed between the two groups (*p* = 0.301, *p* = 0.627, respectively). Treated eyes exhibited significantly higher SFCT, mean PPCT, and TCA values than untreated eyes (*p* < 0.05). See Table 3. See Fig. 5.

**Table 3:** Choroidal thickness, scleral thickness and biomechanical properties among treated and untreated eyes in patients who underwent unilateral corneal cross-linking. Note.

	Untreated eyes in unilateral CXL (*n* = 20)	Treated eyes in unilateral CXL (*n* = 20)	*P* value
Biomechanical properties			
Keratoconus stage, n (%)			0.301
1	12 (60%)	12 (60%)	
2	8 (40%)	6 (30%)	
3	0	2 (10%)	
K mean, mean ± SD	47.68 ± 2.88	48.57 ± 5.01	0.627
Axial length, mean ± SD (mm)	23.74 ± 0.73	23.78 ± 0.66	0.556
IOPg, mean ± SD (mmHg)	10.65 ± 3.03	10.67 ± 3.27	0.962
IOPcc, mean ± SD (mmHg)	13.77 ± 2.23	14.70 ± 2.20	0.065
CH, mean ± SD	8.36 ± 1.77	7.75 ± 1.56	0.255
CRF, mean ± SD	7.14 ± 2.04	6.70 ± 2.09	0.466
Macular choroidal parameters			
SFCT, mean ± SD (µm)	372.85 ± 71.2	434.12 ± 75.03	**0.020**
TCA, mean ± SD (mm^2^)	1.31 ± 0.31	1.43 ± 0.28	**0.042**
SCA, mean ± SD (mm^2^)	0.48 ± 0.12	0.52 ± 0.12	0.412
LCA, mean ± SD (mm^2^)	0.82 ± 0.12	0.91 ± 0.12	0.051
CVI, mean ± SD (%)	0.62 ± 0.02	0.63 ± 0.01	0.421
Peripapillary choroidal thickness			
Mean PPCT, mean ± SD (µm)	211.12 ± 69.49	235.17 ± 73.84	**0.038**
Inferior PPCT, mean ± SD (µm)	196.42 ± 84.33	198.14 ± 91.12	0.609
Superior PPCT, mean ± SD (µm)	229.28 ± 77.80	241.92 ± 83.59	0.117
Nasal PPCT, mean ± SD (µm)	213,71 ± 80.21	230.57 ± 73.61	0.160
Temporal PPCT, mean ± SD (µm)	243.07 ± 64.74	260.07 ± 81.53	0.240
Scleral thickness			
Mean, mean ± SD (mm)	0.46 ± 0.05	0.47 ± 0.04	0.262
Inferior, mean ± SD (mm)	0.47 ± 0.05	0.49 ± 0.05	0.052
Superior, mean ± SD (mm)	0.46 ± 0.06	0.46 ± 0.07	0.630
Nasal, mean ± SD (mm)	0.47 ± 0.05	0.46 ± 0.05	0.646
Temporal, mean ± SD (mm)	0.45 ± 0.05	0.46 ± 0.05	0.223

*CXL *cross-linking, *SD *standard deviation, *SFCT *subfoveal choroidal thickness, *TCA *total choroidal area, *SCA *stromal choroidal area, *LCA *luminal choroidal area, *CVI *choroidal vascularity index, *PPCT *peripapillary choroidal thickness

### Multivariate analysis

Multivariate regression analysis showed that previous CXL was significantly associated with increased SFCT (β = 0.340, *p* = 0.040), while age, CH, AL, KC stage and scleral thickness were not significant predictors. For PPCT and TCA, none of the variables, including previous CXL, reached statistical significance. See Table 4.

**Table 4:** Multivariate regression results

Dependent variable: SFCT		Beta	95% Confidence interval			*P* value		
Age		-0.074			–6.947 to 4.606	0.783		
Previous CXL		0.340			1.298 to 125.688	0.043		
KC stage		0.204			-19.106 to 71.002	0.248		
CH		-0.075			–21.444 to 14.137	0.628		
AL		-0.039			–31.802 to 25.904	0.856		
ST (*mean*)		0.094			–679.976 to 1124.298	0.648		
Dependent Variable: PPCT		Beta	95% Confidence Interval			P value		
Age		–0.072	–5.728 to 3.876			0.697		
Previous CXL		0.286	–13.398 to 94.668			0.135		
KC stage		-0.082	-46.587 to 29.593			0.652		
CH		0.020	–13.886 to 15.470			0.913		
AL		-0.083	–28.186 to 18.165			0.662		
ST (*mean*)		-0.008	–761.397 to 732.156			0.968		
Dependent Variable: TCA		Beta	95% Confidence Interval			*P* value		
Age		0.280	–0.019 to 0.022			0.876		
Previous CXL		0.181	–0.118 to 0.338			0.113		
KC stage		0.265	-0.037 to 0.273			0.132		
CH		-0.096	–0.078 to 0.046			0.599		
AL		-0.267	–0.167 to 0.028			0.157		
Scleral thickness (*mean*)		0.044	–2.786 to 3.515			0.815		

*SFCT *subfoveal choroidal thickness, *PPCT *peripapillary choroidal thickness, *TCA *total choroidal area, *CXL *cross-linking, *CH *corneal hysteresis, *AL *axial length, *ST *scleral thickness

## Discussion

The primary finding of the present study was that SFCT, mean PPCT, and TCA were significantly higher in eyes that had undergone previous epi-off CXL compared to eyes that had not undergone CXL. Similar results were observed when comparing treated and untreated eyes in patients who underwent unilateral CXL. Furthermore, PPE-like alterations were observed exclusively in CXL-treated eyes (16.7%), and previous CXL was the only independent predictor of increased SFCT.

Several studies have demonstrated a significant increase in choroidal thickness in eyes with KC [3–8]. However, findings regarding choroidal changes following CXL remain inconsistent. Nasrollahi et al. reported no significant change in SFCT one month after CXL, whereas Doğanay et al. observed an increase in choroidal thickness three months after surgery [22, 23]. By including eyes treated at least six months prior to imaging, our findings suggest that CXL is associated with choroidal thickening, which may develop progressively over time. These results highlight the need for longitudinal studies to clarify the time course and mechanisms underlying such changes.

Although CXL primarily targets corneal collagen, animal and human studies have shown that riboflavin–UVA cross-linking strengthens scleral collagen, increases mechanical stiffness, and reduces tissue permeability, with variation in effect depending on the scleral region [13, 24, 25]. A thickened and rigid sclera may contribute to choroidal congestion through compression of the vortex vein pathways and may also hinder transscleral macromolecular diffusion, leading to fluid accumulation in the outer choroid and suprachoroidal space [11, 26]. However, no significant differences in anterior scleral thickness were found between the two groups, suggesting that potential functional changes in scleral biomechanical properties or permeability, if present, may occur without measurable structural thickening. Accordingly, our results do not support a direct structural association between scleral remodeling and the choroidal changes observed in treated eyes. It is worth noting that the measurements performed 6 mm posterior to the scleral spur may not adequately represent the biomechanics of the posterior sclera, for which reliable in vivo assessment methods remain limited. Therefore, our findings should be interpreted cautiously, as they provide only indirect evidence of a possible association between CXL and alterations in choroidal biomarkers, while direct evidence of scleral or choroidal remodeling after CXL remains limited. Future studies are warranted to confirm our findings and explore alternative mechanisms, including vascular or inflammatory alterations, that may contribute to the observed findings.

Previous studies have reported a prevalence of PPE in KC ranging between 10.5% and 17.9% [10, 11]. PPE is part of the pachychoroid disease spectrum and is characterized by dilated choroidal vessels and increased choroidal thickness associated with RPE abnormalities [27]. By definition, PPE lacks subretinal fluid and is therefore considered an asymptomatic form fruste of CSC. Long-term follow-up studies indicate that approximately 17% of PPE cases progress to CSC within six years, and some eyes may evolve into other pachychoroid entities such as polypoidal choroidal vasculopathy or pachychoroid neovasculopathy [28, 29]. The pathogenesis is thought to involve choriocapillaris ischemia secondary to thickening of the Haller layer, leading to reduced perfusion of the RPE and outer retina. The increased prevalence of PPE-like changes in KC has been attributed to dysregulation of proteoglycans and glycosaminoglycans. This may reduce transscleral macromolecular diffusion, leading to choroidal thickening and secondary alterations in the RPE and retina [11]. Our findings align with previous reports linking keratoconus to PPE. Furthermore, we observed the exclusive presence of PPE-like changes in CXL-treated eyes in a young cohort of patients with KC. It is worth noting that the lower PPE prevalence of 7.69% observed in our cohort compared with previous studies may be related to the younger mean age of our participants, as younger individuals may have more effective compensatory mechanisms within the RPE and choroidal vasculature that mitigate PPE development.

The exclusive PPE-like anomalies and the greater choroidal thickness observed in the CXL-treated eyes, combined with prior CXL emerging as a predictor of choroidal thickness in our model, collectively support the hypothesis that CXL may be associated with early pachychoroid alterations in an already predisposed population. Nonetheless, plausible confounding factors should not be overlooked; notably, the significant inter-eye difference in CT we found in individuals who underwent CXL unilaterally does not clear the picture for CXL being the sole potential causal factor. A more rapid progression in a keratoconic eye, which would lead to an earlier indication to CXL compared with a fellow clinically stable eye, could be a neglected yet possible confounding element that has to be taken into account, as faster progression of keratoconus itself could be associated with other ocular or systemic factors influencing choroidal thickness.

Corneal biomechanical parameters did not differ significantly between groups, although a trend toward lower CH was observed in the CXL-treated eyes. This finding is consistent with recent meta-analyses indicating that air-puff–derived measurements, such as those provided by the ORA, may underestimate the biomechanical effects of CXL, potentially due to limited sensitivity to subtle post-treatment changes [30]. Employing more advanced biomechanical assessment tools could enhance the detection and understanding of corneal and scleral rigidity alterations following CXL, clarifying their impact on choroidal thickening and PPE formation.

Several limitations of the present study should be acknowledged. The relatively small sample size, though comparable to similar studies, may limit the generalizability of our findings. Further characterization of pachychoroid features in eye with PPE with indocyanine green angiography and OCT-angiography was not performed, which could have been more revelative of vascular changes related to PDS. The evaluation of ocular biomechanics was limited to the ORA, and the inclusion of additional diagnostic devices could provide a more comprehensive characterization of corneal and scleral mechanical properties. Furthermore, the cross-sectional design does not allow the establishment of causal relationships or temporal evolution of the observed changes, and the retrospective nature of patient recruitment may have introduced a selection bias. In addition, a potential indication bias cannot be excluded in this context, as CXL is typically indicated in documented progression of KC; therefore, a more rapidly progressing disease could be associated with other and unknown factors influencing choroidal thickness. A prospective longitudinal approach would be necessary to confirm our findings and to understand better the dynamic relationship between CXL, choroidal thickness, and PPE development in keratoconus.

In summary, we describe measurable and significant differences between untreated and CXL-treated keratoconic eyes, with the latter showing increased choroidal thickness. Also, PPE-like changes were observed exclusively in CXL-treated eyes, and prior-CXL was found to be the only significant predictor of choroidal thickness in our model. Whilst these findings are intriguing and may point to a potential association between CXL and pachychoroid alterations in keratoconic eyes, one must acknowledge that causality cannot be implied as for the intrinsic limits of our study design. Further prospective longitudinal studies with larger cohorts and multimodal assessment are warranted to clarify the mechanisms, time course, and long-term implications of choroidal changes following CXL.

## Supplementary Information

Below is the link to the electronic supplementary material.

Supplementary Material 1

## Data Availability

The data that support the findings of this study are available from the corresponding author upon reasonable request.
