# Supplementary material for: Comparison of choroidal biomarkers and scleral thickness in keratoconus in relation to corneal cross-linking
Source: Graefes Arch Clin Exp Ophthalmol. 2026 Apr 24;264(10):3205–14. doi: 10.1007/s00417-026-07243-y (PMC13635054; doi:10.1007/s00417-026-07243-y)
Supplement: Supplementary file 1 [file 417_2026_7243_MOESM1_ESM.docx]

|  | **Treatment-naive**  **(n=35)** | **Previous CXL**  **(n=30)** | **P value** |
| --- | --- | --- | --- |
| Staging, number (%) |  |  | 0.505 |
| - 1 | 20 (57.1%) | 18 (60%) |  |
| - 2 | 13 (37.1%) | 8 (26.6%) |  |
| - 3 | 2 (5.7%) | 4 (13.3%) |  |
| BCVA, mean ± SD (LogMar) | 0.64 ± 0.32 | 0.51 ± 0.32 | 0.218 |
| IOPg, mean ± SD (mmHg) | 10.22 ± 2.36 | 10.31 ± 2.69 | 0.843 |
| IOPcc, mean ± SD (mmHg) | 13.53 ± 3.68 | 14.13 ± 2.54 | 0.178 |
| CH, mean ± SD | 8.88 ± 2.12 | 8.18 ± 1.65 | 0.237 |
| CRF, mean ± SD | 7.58 ± 1.71 | 6.78 ± 1.68 | 0.213 |
| Axial length, mean ± SD (mm) | 24.23 ± 1.45 | 23.76 ± 0.78 | 0.341 |
| Pachymetry, mean ± SD (µm) | 502.04 ± 32.35 | 458.66 ± 36.78 | **0.001** |
| SFCT, mean ± SD (µm) | 346.56 ± 75.2 | 427.45 ± 76.83 | **0.010** |
| TCA, mean ± SD (mm^2^) | 1.25 ± 0.21 | 1.48 ± 0.31 | **0.035** |
| SCA, mean ± SD (mm^2^) | 0.50 ± 0.16 | 0.54 ± 0.12 | 0.287 |
| LCA, mean ± SD (mm^2^) | 0.80 ± 0.12 | 0.93 ± 0.22 | **0.031** |
| CVI, mean ± SD (%) | 0.63 ± 0.06 | 0.64 ±0.05 | 0.623 |
| MEAN PPCT, mean ± SD (µm) | 203.18 ± 67.35 | 247.32 ± 68.88 | **0.035** |
| MEAN ST, mean ± SD (mm) | 0.46 ± 0.02 | 0.47 ± 0.05 | 0.284 |

***Supplementary Table 1****. Demographic data,* ***c****horoidal thickness, scleral thickness and choroidal biomarkers among the two groups including the left eye in patients for whom both eyes met the inclusion criteria (12 eyes in the treatment-naïve group, 10 in the CXL group)* ***Abbreviations:*** CXL=cross-linking; SD=standard deviation; BCVA=best corrected visual acuity; IOPg=Goldmann intraocular pressure; IOPcc=corneal compensated intraocular pressure; CH=corneal hysteresis; CRF=corneal resistance factor; K mean=mean keratometry. SFCT=subfoveal choroidal thickness; TCA=total choroidal area; SCA=stromal choroidal area; LCA=luminal choroidal area; CVI=choroidal vascularity index; PPCT=peripapillary choroidal thickness; ST=scleral thickness.
